# Calcium pyrophosphate dihydrate deposition disease (CPPD)/Pseudogout of the temporomandibular joint – FNA findings and microanalysis

**DOI:** 10.1186/1742-6413-5-8

**Published:** 2008-04-21

**Authors:** Asghar H Naqvi, Jerrold L Abraham, Robert M Kellman, Kamal K Khurana

**Affiliations:** 1 Department of Pathology, SUNY-Upstate Medical University, 750 East Adams Street, Syracuse, NY 13210, USA; 2 Deptartment of Otolaryngology, SUNY-Upstate Medical University, 750 East Adams Street, Syracuse, NY 13210, USA

## Abstract

We report a case of a Calcium pyrophosphate dihydrate deposition disease (CPPD) presenting as a mass in the parotid and temporomandibular joint (TMJ) that simulated a parotid tumor. A 35 year-old man presented with pain in the left ear area. A CT Scan of the area showed a large, calcified mass surrounding the left condylar head, and extending into the infratemporal fossa. FNA of the mass showed birefringent crystals, most of which were rhomboid with occasional ones being needle shaped, embedded in an amorphous pink substance. Scanning electron microscopy (SEM) with energy dispersive x-ray spectroscopy (EDS) of these crystals showed peaks corresponding to calcium and phosphorus. SEM/EDS is a rapid method of diagnosing calcium pyrophosphate dihydrate deposition disease (CPPD) and an alternative to more commonly used method of special staining of cell block sections coupled with polarizing microscopy.

## Introduction

Calcium pyrophosphate dihydrate deposition disease is characterized by the accumulation of calcium pyrophosphate dihydrate crystals in intra-articular and periarticular tissues. CPPD of the temporomandibular joint is an uncommon entity. It is characterized by the presence of crystal deposits that can lead to the formation of a tumor/mass.

We report a case of TMJ CPPD diagnosed by fine needle aspiration and microanalysis using SEM/EDS. To the best of our knowledge, the cytologic description of CPPD on FNA in TMJ has not previously been reported.

## Case presentation

A 35-year-old man presented to his physician with severe pain in his left ear. Examination showed a possible pustule within the external auditory canal along with TMJ discomfort. He was treated for ear infection but the lesion in the external auditory canal failed to resolve. A subsequent computed tomographic scan revealed a mass in the TMJ area. A more detailed history later revealed that the patient had been having discomfort/pain in the TMJ area for the past 4 years. The patient developed imbalance and tinnitus in the last few months along with hearing loss. An examination of the left parotid showed an area of obvious fullness in the preauricular area. External ears on both sides were normal. The left external auditory canal was tender and showed a small prominence in the anterior part of the canal. The left tympanic membrane was abnormal and showed mild erythema. The CT scans showed a calcified mass surrounding the left mandibular condyle, and extending medially into the infra-temporal fossa. The mass was eroding the floor of the middle fossa and the anterior wall of the epitympanum. A fine needle aspiration (FNA) of the mass was performed in the clinic. Subsequently, the patient underwent resection of the mass and TMJ condylectomy. The mass eroded the zygomatic process of the temporal bone superiorly and extended anterior and medial to the mandibular condyle.

### Pathologic findings

#### Cytopathologic findings

**Figure 1:**
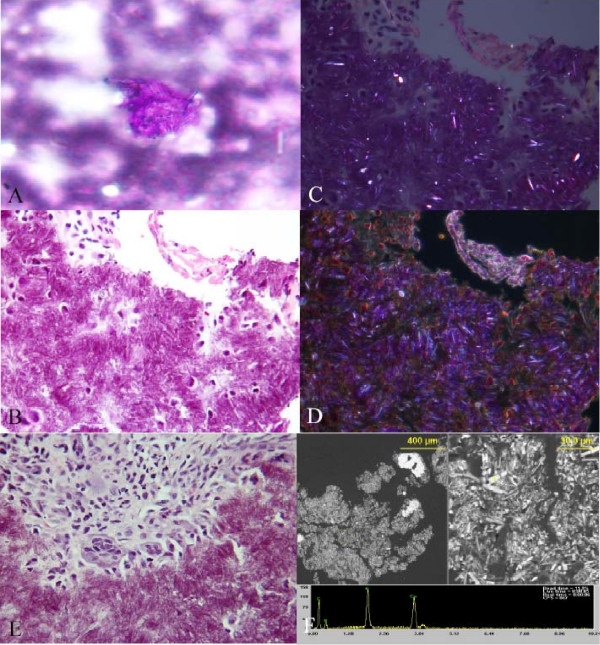
A: Aggregate of crystals on fine needle aspirate, Diff-Quik stain. B: Aggregate of crystals and few mononuclear cells, FNA cellblock, H&E stain. C: Birefringent crystals on polarized light microscopy of same area from cell block. D: Darkfield image showing crystals, same area from cellblock. E: Aggregate of crystals surrounded by mononuclear and multinucleated giant cells in tissue section, H&E stain. F: SEM/EDS of cellblock material, showing crystals at low and higher magnification, and EDS spectrum confirming presence of Ca and P in a single crystal (arrow).

A fine needle aspiration of the mass was performed using a 23-gauge needle after anesthetizing the left parotid area with 2% lidocaine. Smears were made from the aspirate and stained with Diff-Quik and Papanicolaou stain. Multiple aspirations were obtained from different areas of the mass. The aspirate showed abundant extra-cellular crystals, which at places seem to be forming tophi (Figure 1A). The crystals were weakly birefringent, rhomboid with blunt ends, consistent with calcium pyrophosphate crystals (Figures 1B,1C). A few giant cells and mononuclear cells were also identified. The histiocytic cells had abundant coarsely granular-vacuolated cytoplasm with variable number of nuclei. A large number of histiocytic cells had intracytoplasmic crystals in Diff-Quik and to a lesser extent in Papanicolaou stained smears. The cellblock material showed aggregates of crystals, mononuclear cells and few multinucleated giant cells. Focally, the crystal aggregates were dark blue (basophilic). No epithelial cells or any other tissue was obtained. Dark field examination showed abundant crystals (Figure 1D).

#### Surgical pathology

The resected mass consisted of multiple fragments of tan-brown soft tissue from 0.5 to 3.0 cm (in aggregate 5.0 × 5.5 × 0.7 cm) with a chalky white covering. The resected portion of the condyle was 1.5 × 1.5 × 1.2 cm. The H&E stained section of the fragments showed nodular aggregates of crystals, surrounded by dense collagenous fibrosis (Figure 1E). Areas of chondroid metaplasia were seen interspersed with aggregates of crystals. A large number of multinucleated giant cells were seen in the background along with a lymphoplasmacytic infiltrate (Figure 1E). Focal areas of calcification were present. The crystals showed similar morphology and birefringence as seen in the FNA specimen. The section of the condylar bone was unremarkable.

#### Electron Microscopic findings

The freshly cut surface of the paraffin block from the FNA was directly examined with SEM/EDS using the variable pressure mode to allow examination of such non-conductive samples. The crystals demonstrated peaks for calcium and phosphorus. The crystals were present in groups/tophi and were mostly rhomboid with few needle shaped ones intermingled (Figure 1F). The crystals ranged from 2 – 25 um length and 0.5 – 5 um diameter.

## Discussion

CPPD is a rare, benign crystalline arthropathy of unknown cause. It is presumed to result from a disturbance of phosphate metabolism. Pritzker et al in 1976 first described a case of pseudogout in the TMJ but the term CPPD was first proposed by Ryan and McCarty in 1985[1]. It is usually a monoarticular condition characterized by crystal deposition of calcium pyrophosphate in synovial membranes and joint cartilages. However, Chuong and Piper[2] reported the rare occurrence of bilateral pseudogout of the TMJ. Neoplasms of the TMJ are rare but can be a diagnostic challenge[3]. CPPD exhibits a range of clinical presentations, from absence of symptoms to severely destructive arthropathy or conditions simulating a neoplasm.

Two main forms of CPPD have been described – common/diffuse and tumoral. The common type of CPPD (pseudogout) usually affects larger joints and often follows trauma, surgery, or ischemic heart disease. The tumoral type mainly affects the TMJ, cervical spine and hand. The main differential diagnosis includes tophaceous gout, tumoral calcinosis, synovial chondromatosis[4], and benign and malignant tumors.

The crystals of uric acid in gout are water-soluble (best preserved with alcohol fixation); needle shaped and demonstrates negative birefringence with polarized light, whereas the crystals of pseudogout have blunt/squared ends with weak birefringence. The crystals of CPPD are 2–40 micrometer, typically rhomboid shaped but small-long rods and even squares may be seen[5]. Areas of chondroid metaplasia and atypical chondrocytes in CPPD may raise the suspicion for chondroma[6] or chondrosarcoma[3]. Calcified chondroma contains calcium hydroxyapatite where as tumoral calcinosis is characterized by calcium phosphate deposits[6]. Both these deposits are amorphous and do not display birefringence [6].

The role of FNA in diagnosis of CPPD has been described in the neck[7,8] vertebral body[9], other joints (ankle, metatarso-phalangeal, knee)[10], surrounding soft tissue[10], and paraischial soft tissue mass[11]. (Table 1) The aspirated crystals may be intracellular or extracellular [7-10]. However, in our case the crystals were predominantly extracellular. Our findings of weakly birefringent crystals is similar to that of Rothschild et al[10]. H&E stained histologcal sections may not allow proper evaluation of birefringence properties of the crystals in the lesions of CPPD and gout. Shidham et al evaluated the usefulness of nonaqueous alcoholic eosin staining (NAES) method followed by polarizing microscopy in the detection of crystals of gout, CPPD and tumoral calcinosis. They found that NAES stained section of CPPD showed positive birefringence as compared to negative birefringence of gout in a significant number of cases. NAES method can be a useful adjunct to regular H&E stained section in the detection of different types of crystals[12]. Previous reports have described the crystals of CPPD as rectangular, needle or rhomboid. In our case the crystals were predominantly rhomboid. CPPD crystals were seen predominantly on Diff-Quik stain, as also reported by Pakzad et al[9] and Biankin et al[8]. Presence of few crystals in Papanicolaou stain in our case may be attributed to fixation of smears in acid-alcohol, which may cause dissolution of crystals[9]. In contrast, Allen et al[7] have described yellowish-orange crystals on Diff-Quik stained smears. Multiple factors including stain intensity, interference with other chemicals or a different type may have resulted in the yellowish-orange crystals. Also, no microanalysis was performed in that case, which could have further helped in characterization of the crystals.

**Table 1:** 

Authors	Age (year)/Sex	Location	Cytopathology	Histopathology	SEM/EDS
Rothschild et al [10]	67/M, 55/M, 52/M, 51/M, 43/M, 53/M, 61/M	Ankle, MTP, Knee, Soft Tissue	Weakly positive birefringent crystals	ND	ND
Allen et al [7]	73/F	Neck	Numerous macrophages with intracytoplasmic yellowish-orange rhomboid crystals, few extracellular crystals	ND	ND
Lambrecht et al [11]	76/F	Paraischial soft tissue mass	Clusters of crystals, needle shaped to rectangular, chondrocytes with atypia, few foamy histiocytes, rare multinucleated giant cells	Multinodular chondromyxoid lesion, crystalline material, multinucleated giant cells	ND
Pakzad et al [9]	73/M	Neck	Abundant neutrophils & small birefringent rhomboid crystals	ND	ND
Biankin et al [8]	75/M	Neck	Histiocytes with numerous intracellular and scattered extracellular crystals	ND	ND

ND: Not Done

The cell block can be used for histopathological findings and also for SEM/EDS. Our findings of aggregates of crystals with basophilic appearance on cell block was similar to that previously reported by Pakzad et al[9]. Our histopathological findings were characteristic of CPPD and comprised of a multinodular chondromyxoid lesion, with intervening fibrous bands and accompanying multinucleated giant cells and other chronic inflammatory cells[6].

CT scan is considered to be the best imaging modality for the diagnosis of CPPD[6]. The CT scan in our patient showed a large mass eroding the floor of the middle fossa, anterior wall of the epitympanum and a malignancy was initially suspected. CPPD of the TMJ often leads to periarticular and intra-articular calcification and is commonly associated with pain, swelling, trismus, and hearing loss. Our patient had severe pain in his ear that was clinically thought to be due to infection but failed to resolve with antibiotic treatment. The diagnosis of CPPD should be considered in patients with pain or mass in the TMJ area. The treatment of CPPD is surgical with a high chance of recurrence[3].

A detailed literature search revealed that SEM/EDS has been used in characterizing the crystals of CPPD in a few cases. Dijkgraaf et al[13] examined CPPD cases using transmission electron microscopy and found numerous extra – as well as intracellular crystals and crystal shaped spaces in the chondrocytes. Strobl et al[1] used infrared spectrophotometry to analyze the crystals. The crystals in our case demonstrated peaks for calcium and phosphorus on SEM/EDS, which is consistent with the findings of CPPD.

In summary, FNA findings coupled with SEM/EDS when available can provide a rapid and accurate diagnosis of CPPD of TMJ and an alternative to other ancillary techniques including polarizing microscopy along with special stains like NAES.

## Authors' contributions

The cytology diagnosis was made by Dr. Asghar H Naqvi and Dr. Kamal K Khurana. Dr. Jerrold L Abraham rendered the Scanning Electron Microscopy report. Dr. Robert M Kellman clinically evaluated the patient. Dr Asghar H Naqvi, Dr Jerrold L Abraham and Dr Kamal K Khurana equally contributed in drafting and designing the manuscript. All the authors read and approved the final manuscript.
